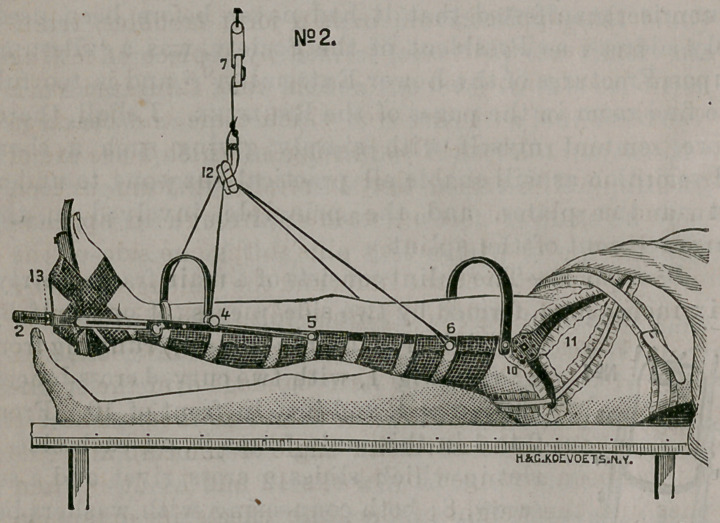# General Lower Extremity Splint

**Published:** 1881-11

**Authors:** A. Clendinen

**Affiliations:** Fort Lee, N. J.


### GENERAL LOWER EXTREMITY SPLINT.—EXTENSION AND COUNTER EXTENSION BY WEIGHT OF LIMB, WITH CAPACITY FOR FIXATION.

By A. CLENDINEN, M.D., Fort Lee, N. J.

I herewith submit plates illustrative of my splint, which was shown at the annual meeting of the Bergen County District Society in April, last, and also exhibited, and its suspension by tripod demonstrated, at the annual meeting of the New Jersey State Medical Society. It was approved by all who were present, and great admiration was expressed at the simplicity of the device, and much surprise manifested that it had never before been used. My address, as President of the Society, was a "Resume upon Fractures of the Lower Extremity,"* and is too full to find room in the pages of the Register. I shall, therefore, content myself with simply giving such a short description as will enable all practical surgeons to understand the plates, and the principle involved in the arrangement of the splint.

*Description.*—The splint consists of a main frame, thirty-six inches long, formed by two side pieces (of one-half by one eighth inch, iron or steel,) running from 2 to 10, Fig. 1, with two curved cross braces, as seen at 4, and just in front of 10. From 2 to 4 in this main frame is, on each side, a slot in which slides a cross rivet and a set screw, 8; both connecting, with washers between, the outside pieces, 3, to the inside or foot piece, 1. This slot is eight inches long, so that the splint can be used for almost any adult limb. On the outside piece, 3, at 5, is a knob for the attachment of a rope or cord, to pass forward and under the pulley, 4, (which revolves upon a rivet in the brace) and thence through the block, 12, back to the knob, 6, on the main frame. At 12 the angle of suspension can be set as

*Not yet in print by the Society.

thought proper, and from 12 to the ceiling, or other attachment, the degree of elevation can be regulated by the block, 7, as shown in Fig. 2.

Irons 9, Fig. 1, are attachable by a screw bolt at the end of the main frame, and their angle is regulated by a pin in the holes at 10. After setting the pin and tightening the set bolt, the horizontal position will be obligatory, but by the loosening of the same—as in tibia or fibular fractures—with counter extending plasters from below the knee, section 10 resolves itself into a mere hinge of even pelvic bearing, allowing a free and harmless set up by the patient. The pieces forming the triangle at the hip joint section, 11, Fig. 1 and 2, are bent at angles to suit the flesh lines, and are of different length and shape from those used on the inside, which are twisted to show flat surfaces toward the pubes and the perineum, but these pieces can be changed to either side, thus allowing the splint to be applied to either limb. Particular attention is drawn to the fact that not only can the hip joint section, 11, be most comfortably fixed and controlled, (the lack of which ability has often been realized in neck fractures, trochanteric impactions, etc.,) but that by regulation of the angle of the rubber bearing, at the end of 9, by the set of pin at 10, the line of counter extension can effect inversion or eversion of the limb, which, in some of the shaft fractures, is, with proper elevation, very desirable. The peculiarities of the foot piece are, that, in addition to the slot in the outside iron and set screw at 13, Fig. 2, by which the foot may be raised or lowered and the limb inverted or everted, by the above and below rectangular slots such attachments and comfortable bearing can be had above the heel and over the instep as to make unnecessary the attachment of plasters, which, at any rate, are comparatively useless in fractures of the lower third of the tibula and fibula.

*Application.*—Whilst the limb is resting on mattress, 9, irons with rubber tubing bearing upon cushions (hair), are adjusted to the parts, and abdominal band and cushion in connection applied; and in hip joint troubles, triangle, 11, has been additionally fixed by a piece of felting or paste board, moulded to section, and saturated with starch, plaster or silicates, over which come the bearings of the iron and rubber. The bandages (which I use are of hair cloth, for the reason that it retains its form, is cool, clean and capable of being washed through in applications to limb,) are attached by pins to the one side of the splint, which is set over the limb, and the bandages, one by one, are drawn through and pinned to suit the limb lines; next, the connection at 10 is made by the screw bolt, and the foot piece is slid up; the hair bandage over the stocking is drawn out through the slots and pinned. The set screw, 8, is kept tight until the angle of the cord and elevation has been arranged by blocks 12 and 7. It is then loosened and the weight of the limb effects its extension by the carrying forward of the foot piece, and counter extension by back pressure of the main frame, at 10, on the pelvic bearings. If the weight of the limb is not sufficient, and no previous traction has been made, or, in some cases of oblique fracture, extra pressure can, for the moment, be made upon the splint, and the screw set at 8 until such time as the extra muscular spasm shall have subsided, or if the surgeon in any cases should be afraid of too much extension, he can, at the proper extension, effect fixation by tightening the set screw, 8, and he can constantly compare the measurement of the limbs.

The pelvic bearings, in effecting counter extension, are of self adjustment, do not gall, and by the open angles circulation cannot be interfered with. The pressure can be regulated by the angle at 12, and by elevating or depressing the head of the bedstead.

My experience during the late civil war decided me as being adverse to flexion and as in favor of suspension, and since the war this opinion has been confirmed. In reference to suspension, the trouble has been that no apparatus was capable of uniform extension, and none capable of counter extension, therefore many surgeons have imagined themselves forced, in various instances, to lay the limb of patient upon the bed, not only to his discomfort but to his injury; because, if in the case of an oblique fracture, it would be impossible for him to move his body (and this he will do) without disturbing the point of union, and no bandages can be borne taut enough at the foot, groin or elsewhere to obviate friction and to prevent displacement.

I hope the day will, ere long, come when the professors of clinical surgery will be expected to accurately measure limbs, both before and after union, so that results may be fairly compared. So far as my experience and my comparisons go, this splint has been found capable of the best results; I shall, however, welcome and use with great pleasure anything that may afford results superior to this.

## Figures and Tables

**No 1. f1:**
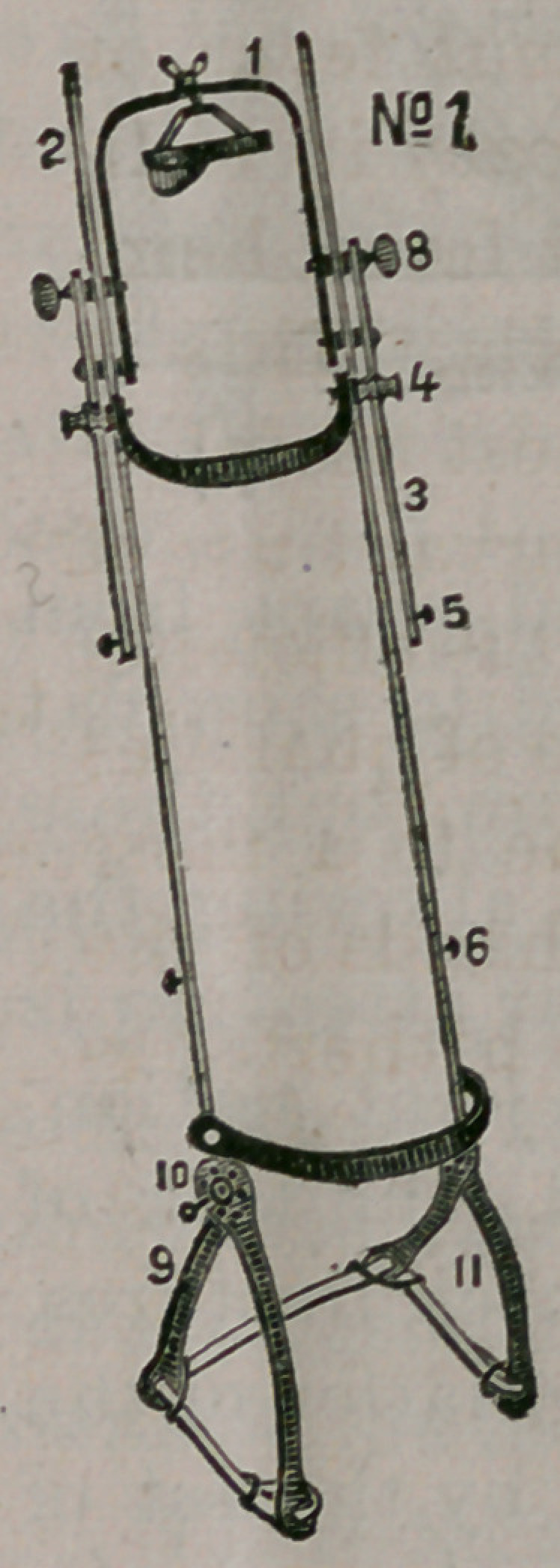


**No 2. f2:**